# Iatrogenic infection in dermoid cysts of the floor of the mouth

**DOI:** 10.1590/S1808-86942011000500023

**Published:** 2015-10-22

**Authors:** Thiago de Santana Santos, Ana Cláudia Amorim Gomes, Riedel Frota, Emanuel Dias de Oliveira e Silva, Paulo Ricardo Saquete Martins Filho, Emanuel Sávio de Souza Andrade

**Affiliations:** 1 Specialist in buccomaxillofacial surgery and trauma, Brazilian College of Buccomaxillofacial Surgery and Traumatology. Master's degree in buccomaxillofacial surgery and trauma, Pernambuco Dentistry School (FOP), UPE; 2 Doctoral degree in buccomaxillofacial surgery and trauma, Pernambuco Dentistry School (FOP), UPE. Adjunct professor, Pernambuco Dentistry School (FOP), UPE; 3 Doctoral degree in buccomaxillofacial surgery and trauma, Pernambuco Dentistry School (FOP), UPE. Staff of the residency program in buccomaxillofacial surgery and trauma, Oswaldo Cruz University Hospital (HUOC), UPE; 4 Specialist in buccomaxillofacial surgery and trauma. Head of the residency and specialization program in buccomaxillofacial surgery and trauma, Oswaldo Cruz University Hospital (HUOC), UPE; 5 Master's degree in health sciences, Graduate Nucleus in Medicine, Sergipe Federal University (UFS). Substitute professor of oral pathology, Sergipe Federal University (UFS); 6 Doctoral degree in oral pathology, Rio Grande do Norte Federal University (UFRN). Adjunct professor of oral pathology, Pernambuco Dentistry School (FOP), UPE

**Keywords:** dermoid cyst, iatrogenic disease, mouth floor, surgery, oral

## INTRODUCTION

Dermoid cysts are infrequent cystic tumors with an epidermal epithelium lining and structures such as hair follicles, sweat glands, and sebaceous glands. These cysts are considered true teratomas, and their origin may be congenital or acquired. The incidence is highest in male or female individuals aged from 15 to 35 years^1^.

## CASE REPORT

**Figure 1:**
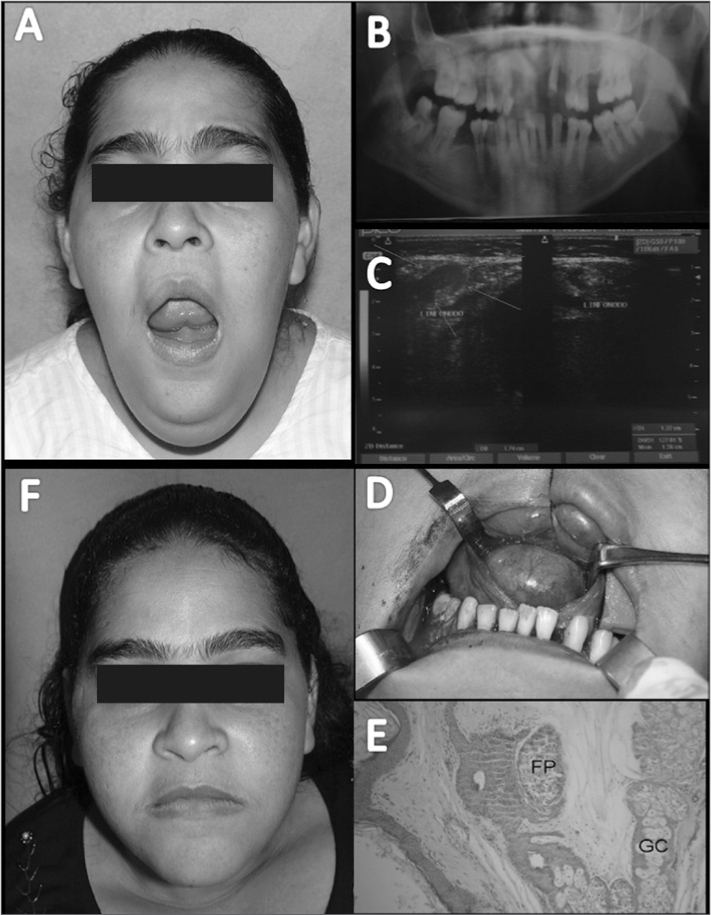
Dermoid cyst - A. Bilaterally enlarged submentual and submandibular mass resulting in a double chin, limited opening of the mouth, and elevated floor of the mouth; B. Panoramic radiograph of the jaws; C. Ultrasound - arrows show a suggested cyst with a semiliquid content within the fascial spaces of the muscles in the floor of the mouth; D. Surgical removal of the lesion through an intraoral approach; E. Histopathology. Note the wall of the cyst consisting of fibrous connective tissue, skin elements such as a hair follicle (PF), and sweat glands (SG) (H.E. 100x); F. Postoperative aspect after 1 year and 6 months.

A female patients aged 34 years reported a progressive bilateral growth in the submentum and submandibular regions during the past 6 years. She added that it had worsened within the past seven days following needle aspiration biopsy in the floor of the mouth (Figure 1A). The physical examination revealed fever, dysphonia, dysphagia, and dyspnea. The patient was admitted into the hospital for surgical drainage through an intraoral approach; the drained material consisted of pus and a cyst containing hairs and a yellowish viscous material similar to keratin. A panoramic radiograph of the jaws revealed an extensive carious lesion with pulp involvement in unit 48, which suggested an odontogenic infection (Figure 1B). Ultrasound showed a well-defined regularly contoured ecogenic mass in the submentum, suggesting a cyst with viscous liquid content (Figure 1C). The hypothesis was a dermoid cyst. Under general anesthesia, the cyst was enucleated in an intraoral approach (Figure 1D). Histopathology confirmed the hypothesis (Figure 1E). The tumor has not recurred 1 year and 6 months after surgery (Figure 1F).

## DISCUSSION

Dermoid cysts are generally asymptomatic slow-growing tumors of varying size. The anatomical site is critical for its clinical presentation. Lesions above the geniohyoid muscle may enlarge the sublingual region, while those below this muscle may enlarge the submentual region and generate a double chin aspect^2^, ^3^, ^4^. In the present case, although the lesion was above the geniohyoid muscle, the initial infection and concomitant obesity of the patient resulted in a double chin, which made for an atypical presentation of a tumor located sublingually.

The differential diagnosis of dermoid cysts in the floor of the mouth is made with several other lesions, such as ranula, obstructed submandibular/sublingual ducts, thyroglossal duct cysts, odontogenic infection, benign and malignant tumors, and even excessive fat in the submentual region. Besides the clinical evaluation, computed tomography, ultrasound, and biopsies may be used to clarify the diagnosis.^1^ Upon careful palpation, dermoid cysts fluctuate or feel similar to dough; ultrasound imaging adequately supplements the physical examination. Thus, aspiration biopsies, which are not recommended in the literature for the diagnosis of dermoid cysts, may be avoided, and may be considered an iatrogenic procedure in this situation. An altered physical status and the presence of poor dental health led to an initial diagnosis of odontogenic infection involving the primary fascial spaces.

Therapy consists of surgical removal of lesions, either extra- or intraorally, depending on the site and number of tumors^2^,^5^. In the present case, removal was done through an intraoral approach as the lesion was located above the geniohyoid muscle.

## FINAL COMMENTS

A careful physical examination may avoid iatrogenic procedures when a dermoid cyst is suspected; these lesions generally have well-defined features when located in the floor of the mouth.
